# Detection of anatid herpesvirus 1 *gC *gene by TaqMan™ fluorescent quantitative real-time PCR with specific primers and probe

**DOI:** 10.1186/1743-422X-7-37

**Published:** 2010-02-13

**Authors:** Qing Zou, Kunfeng Sun, Anchun Cheng, Mingshu Wang, Chao Xu, Dekang Zhu, Renyong Jia, Qihui Luo, Yi Zhou, Zhengli Chen, Xiaoyue Chen

**Affiliations:** 1 Avian Disease Research Center, College of Veterinary Medicine, Sichuan Agricultural University, Yaan 625014, China; 2 Key Laboratory of Animal Disease and Human Health of Sichuan Province, Yaan 625014, China; 3 Epizootic Diseases Institute of Sichuan Agricultural University, Yaan, Sichuan, 625014, China

## Abstract

**Background:**

Anatid herpesvirus 1 (AHV-1) is known for the difficulty of monitoring and controlling, because it has a long period of asymptomatic carrier state in waterfowls. Furthermore, as a significant essential agent for viral attachment, release, stability and virulence, *gC *(*UL44*) gene and its protein product (glycoprotein C) may play a key role in the epidemiological screening. The objectives of this study were to rapidly, sensitively, quantitatively detect *gC *gene of AHV-1 and provide the underlying basis for further investigating pcDNA3.1-gC DNA vaccine in infected ducks by TaqMan™ fluorescent quantitative real-time PCR assay (FQ-PCR) with pcDNA3.1-gC plasmid.

**Results:**

The repeatable and reproducible quantitative assay was established by the standard curve with a wide dynamic range (eight logarithmic units of concentration) and very good correlation values (1.000). This protocol was able to detect as little as 1.0 × 10^1 ^DNA copies per reaction and it was highly specific to AHV-1. The TaqMan™ FQ-PCR assay successfully detected the *gC *gene in tissue samples from pcDNA3.1-gC and AHV-1 attenuated vaccine (AHV-1 Cha) strain inoculated ducks respectively.

**Conclusions:**

The assay offers an attractive method for the detection of AHV-1, the investigation of distribution pattern of AHV-1 in vivo and molecular epidemiological screening. Meanwhile, this method could expedite related AHV-1 and gC DNA vaccine research.

## Background

Anatid herpesvirus 1 (AHV-1) infection alternatively known as duck virus enteritis (DVE), or duck plague (DP), is one of the most widespread and devastating diseases of waterfowls in the family Anatidae[1]. As an acute and contagious herpesvirus, AHV-1 can infect ducks, geese, and swans of all ages and species[2]. Since the first outbreak in the Netherlands in 1923, AHV-1 had a dramatic impact on international trade of waterfowls and waterfowl products throughout the world [3-5]. Like other herpesviruses, AHV-1 can be carried and periodically shed by recovered birds from the disease. Moreover, the reactivation of latent AHV-1 may threaten domestic and migrating waterfowls populations[6]. AHV-1 has already become an important potential risk factor for waterfowls health.

As a significant agent of AHV-1, *gC *(*UL44*) gene has seldom been reported about the research of its molecular biology, and its research level fall behind relatively in other herpesviruses[7]. Although gC is nonessential component for the viral replication, its protein product (glycoprotein C) has several important biological functions. As a multifunctional glycoprotein in Alphaherpesvirinae, glycoprotein C involves in viral attachment, release, stability, virulence and other functions [8-14]. Being situated on the envelope surface of mature virus particles, glycoprotein C contains many antigen determinants, and can adequately induce immune response [15-18]. Some DNA vaccines based on gC gene from other kinds of herpesviruses immunized in mice or other relative animals could receive good immune responses and protective efficacy [19-23], while the biological functions of AHV-1 glycoprotein C and DNA vaccine based on AHV-1 gC have not been reported. In this study, pcDNA3.1-gC plasmid is not only used as standard DNA to develop a standard curve for TaqMan™ FQ-PCR but also as a DNA vaccine to inoculate ducks.

Many diagnosis and detection methods about AHV-1 have been reported in a long time, such as epidemiological information, viral isolation and immunological methods [24-28]. These tests are laborious and time-consuming resulting from requiring strict operation. Thus, these methods can not be used to direct detection. In addition, the reliable diagnosis is difficult to obtain from mixed or secondary infected waterfowls. AHV-1 is difficult to be monitored and controlled because it has a long period of asymptomatic carrier state in waterfowls[29]. It is usually detected only during the intermittent shedding period of the virus. Thus, how to sensitively detect AHV-1 has become a significant factor from infected waterfowls. PCR is a useful tool with high sensitivity for detecting nucleic acids of virus from the ducks [30-33]. However, the traditional PCR assays still had some flaws, such as poor performance in quantitation and a relative waste of time. It is not suitable for large-scale applications.

In recent times, a more sensitive, time-saving and advanced method has emerged in the field, which is fluorescent quantitative real-time PCR (FQ-PCR). This technology accurately quantifies target DNA in a given sample and then could accurately detect viral loads in clinical samples[34]. Yang and Guo have reported the detection of AHV-1 with FQ-PCR method [35,36]. FQ-PCR based on TaqMan™ technology provides certain advantages including high sensitivity, high specificity, and reproducibility, and has been widely used to quantify the copies of viral genomic after optimization [37-44]. In this study, the developed FQ-PCR method was extremely valuable for AHV-1 detection. Moreover, the results provide some interesting basic data that may be beneficial to further investigate pcDNA3.1-gC DNA vaccine in vivo in ducks.

## Results

### Development and optimization of a TaqMan™ FQ-PCR

Final concentrations of primers each of 0.5 μmol/L and probe of 0.25 μmol/L were selected, and the optimized annealing temperature was 53°C. The combination of primers, probe and annealing temperature was used for subsequent experiments.

### Standard curve establishment

**Figure 1:**
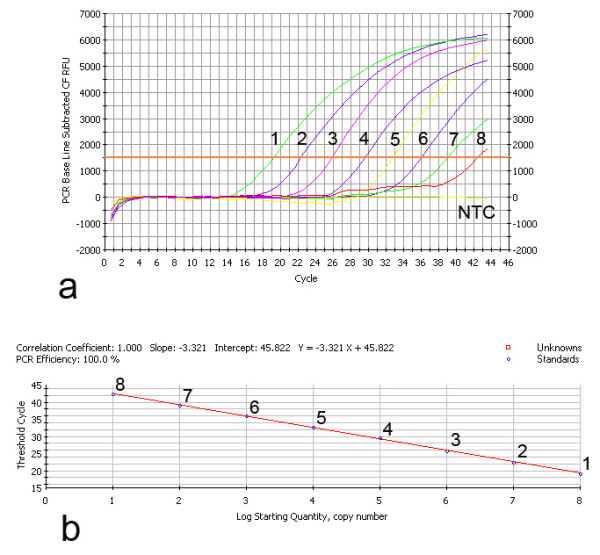
**The amplification curves (Figure 1.a.) and standard curve (Figure 1.b.) of the TaqMan™ FQ-PCR detection**. Ten-fold dilutions of standard DNA ranging from 1.0 × 10^8 ^to 1.0 × 10^1 ^copies/reaction were used (1-8), as indicated in the x-axis, whereas the corresponding Ct values are presented on the y-axis. The correlation coefficient and the slope value of the regression curve were calculated and indicated.

The amplification curves (Figure 1.a.) and standard curve (Figure 1.b.) of the TaqMan™ FQ-PCR were generated by using the 10-fold dilutions of pcDNA3.1-gC, which has already known its copies to undertake FQ-PCR reaction under optimum conditions with the iCycler IQ Detection System. The curve covered a dynamic range of eight log units of concentration and displayed a clear linear relationship with a correlation coefficient of 1.000 and high amplification efficiency (100%). By using the following formula, we were able to quantify the amount of unknown samples: Y = -3.321X + 45.822 (Y = threshold cycle, X = log starting quantity).

### Amplification sensitivity, specificity, repeatability and reproducibility

**Figure 2:**
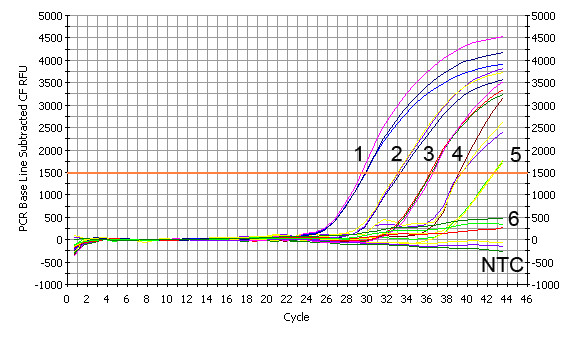
**The sensitivity of TaqMan™ FQ-PCR detection**. Ten-fold serial dilutions of AHV-1 standard template were used (1-6), 1.0 × 10^5^-1.0 × 10^0 ^copies/reaction of AHV-1 standard template. As shown in the figure, the detection limit for the assay was 1.0 × 10^1 ^copies.

Ten-fold dilution series of pcDNA3.1-gC standard DNA (from 1.0 × 10^5 ^to 1.0 × 10^0 ^copies/reaction) were tested by the established FQ-PCR assay to evaluate the sensitivity of the system, the mean threshold cycle (Ct) values were 29.60, 33.10, 36.43, 39.30, 42.57 and N/A respectively. The results showed that the assay could detect down to 1.0 × 10^1 ^copies per reaction (Figure 2.). All liver samples were retested positive for AHV-1 from infected ducks with the established FQ-PCR assay, it indicated that this method was sensitive for clinical cases. Comparisons were made between the established FQ-PCR method and conventional PCR method by using 10-fold dilutions of viral DNA from infected allantoic fluid to calculate the end-point sensitivity of each assay. The results showed that the established FQ-PCR could detect viral DNA down to dilutions of 2.730 × 10^1^, while the dilutions of only 2.730 × 10^4 ^for conventional PCR.

**Figure 3:**
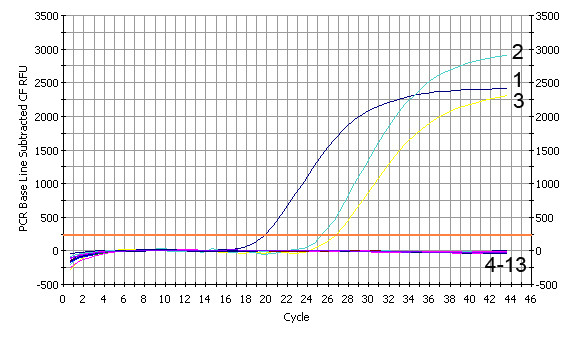
**The specificity of TaqMan™ FQ-PCR detection**. The pcDNA3.1-gC (1), AHV-1 Cha (2), AHV-1 Chv (3), gosling new type viral enteritis virus (4), duck hepatitis virus type1 (5), duck adenovirus (6), goose parvovirus (7), Marek's disease virus (8), *Pasteurella multocida *(5:A) (9), *Escherichia coli *(O78) (10), *Salmonella enteritidis *(No. 50338) (11), the liver DNA of the healthy duck (12) and NTC (13) were tested to evaluate the specificity of the assay by FQ-PCR.

The specificity test showed that pcDNA3.1-gC, AHV-1 attenuated vaccine (AHV-1 Cha) strain virus and AHV-1 virulent (AHV-1 Chv) strain virus were found positive for AHV-1 by the established FQ-PCR assay, while the bacteria, remaining viruses including negative control (liver sample of the healthy duck) were negative (Figure 3.). The results were confirmed by gel electrophoresis, there was a band of the expected size (78 bp) observed exclusively from samples of pcDNA3.1-gC, AHV-1 Cha and AHV-1 Chv. It indicated that the established FQ-PCR assay was highly specific.

In the intra-assay and inter-assay, the mean Ct values and standard deviations (SD) values were calculated. As shown in Table 1, the coefficient of variation (CV) values ranged from 0.44% to 2.03%, indicating that this assay was highly repeatable and reproducible.

**Table 1:** Intra-assay and inter-assay of the TaqMan™ FQ-PCR assay

Variation	Copies of standard	Crossing point		
		
		Mean	**SD**^**a**^	**CV (%)**^**b**^
Intra-assay	1.00E+08	19.73	0.15	0.77
	1.00E+07	23.10	0.20	0.87
	1.00E+06	26.37	0.29	1.09
	1.00E+05	29.63	0.21	0.70
	1.00E+04	33.07	0.31	0.92
	1.00E+03	36.33	0.31	0.84
	1.00E+02	39.50	0.17	0.44
	1.00E+01	42.67	0.32	0.75
Inter-assay	1.00E+08	19.70	0.28	1.41
	1.00E+07	23.09	0.47	2.03
	1.00E+06	26.31	0.32	1.23
	1.00E+05	29.59	0.42	1.42
	1.00E+04	33.04	0.34	1.03
	1.00E+03	36.35	0.41	1.14
	1.00E+02	39.52	0.43	1.10
	1.00E+01	42.70	0.41	0.97

Repeatability and reproducibility (R & R) of the TaqMan™ FQ-PCR assay.

^a ^Standard deviation.

^b ^Coefficient of variation.

### Detection of AHV-1 *gC *gene in samples for practical applications

AHV-1 gC gene and viral load quantification demonstrated that the AHV-1 gC copies of each sample could be calculated by using the Ct value determined from the standard curve. As shown in Table 2, AHV-1 gC can be detected in all analyzed tissues at 1 hour postinoculation. gC copies of all tissues reached a peak at 1 hour postinoculation in gC DNA vaccine-inoculated ducks, while the copies of most tissues (other than kidney) reached a peak at 4 hours postinoculation in AHV-1 Cha strain-infected ducks. The concentration of nucleic acid in DNA vaccine-inoculated ducks maintained 10^7 ^copies/g level at 4 weeks postinoculation. The copies of the liver, spleen and thymus were more than other tissues in gC DNA vaccine-inoculated ducks, while the copies of the duodenum and rectum were relatively low in AHV-1 Cha strain-infected ducks.

**Table 2:** Mean AHV-1 gC copies and viral loads in different samples for practical applications

Samples (lg copies/g)	Groups	1 h	4 h	8 h	12 h	1 d	3 d	5 d	7 d	2 wk	4 wk
Liver	1	9.38 ± 0.23	9.15 ± 0.15	8.93 ± 0.18	8.62 ± 0.09	8.41 ± 0.19	8.18 ± 0.08	8.06 ± 0.20	8.03 ± 0.12	8.03 ± 0.25	7.98 ± 0.14
	2	8.45 ± 0.01	8.62 ± 0.13	8.58 ± 0.08	8.37 ± 0.07	8.23 ± 0.10	7.95 ± 0.19	7.72 ± 0.16	7.57 ± 0.16	7.28 ± 0.14	6.87 ± 0.19
Pancreas	1	8.32 ± 0.03	8.21 ± 0.07	8.13 ± 0.04	8.01 ± 0.12	7.95 ± 0.09	7.87 ± 0.05	7.73 ± 0.17	7.56 ± 0.07	7.42 ± 0.15	7.35 ± 0.05
	2	8.16 ± 0.16	8.38 ± 0.10	8.22 ± 0.14	8.08 ± 0.08	8.00 ± 0.11	7.98 ± 0.06	7.86 ± 0.14	7.61 ± 0.18	7.2 ± 0.03	6.69 ± 0.05
Spleen	1	9.23 ± 0.11	9.06 ± 0.06	8.75 ± 0.10	8.52 ± 0.04	8.32 ± 0.16	8.26 ± 0.14	8.15 ± 0.18	8.12 ± 0.24	7.94 ± 0.08	7.87 ± 0.17
	2	9.26 ± 0.08	9.49 ± 0.06	9.21 ± 0.13	8.92 ± 0.16	8.75 ± 0.20	8.51 ± 0.13	8.33 ± 0.07	8.07 ± 0.81	7.68 ± 0.13	7.53 ± 0.12
Kidney	1	8.47 ± 0.02	8.36 ± 0.05	8.24 ± 0.14	8.13 ± 0.13	7.94 ± 0.11	7.62 ± 0.05	7.48 ± 0.18	7.31 ± 0.27	7.33 ± 0.13	7.29 ± 0.11
	2	8.41 ± 0.13	8.33 ± 0.17	8.23 ± 0.08	8.05 ± 0.15	7.97 ± 0.04	7.82 ± 0.09	7.79 ± 0.11	7.58 ± 0.14	6.96 ± 0.12	6.84 ± 0.06
Lung	1	8.89 ± 0.07	8.77 ± 0.10	8.44 ± 0.14	8.14 ± 0.11	8.02 ± 0.05	7.88 ± 0.18	7.51 ± 0.21	7.50 ± 0.16	7.42 ± 0.11	7.30 ± 0.16
	2	8.34 ± 0.03	8.58 ± 0.03	8.42 ± 0.17	8.21 ± 0.04	8.14 ± 0.16	7.86 ± 0.13	7.82 ± 0.18	7.62 ± 0.21	7.35 ± 0.21	7.11 ± 0.15
Thymus	1	9.2 ± 0.16	9.04 ± 0.11	8.86 ± 0.18	8.52 ± 0.05	8.4 ± 0.19	8.23 ± 0.15	8.11 ± 0.17	8.03 ± 0.01	7.95 ± 0.05	7.84 ± 0.08
	2	9.27 ± 0.14	9.41 ± 0.18	9.22 ± 0.06	8.94 ± 0.20	8.72 ± 0.13	8.38 ± 0.07	8.04 ± 0.17	7.77 ± 0.05	7.56 ± 0.14	7.39 ± 0.15
Heart	1	8.68 ± 0.07	8.59 ± 0.14	8.44 ± 0.17	8.20 ± 0.03	8.13 ± 0.02	8.07 ± 0.13	8.02 ± 0.25	7.94 ± 0.17	7.82 ± 0.22	7.76 ± 0.14
	2	9.06 ± 0.05	9.14 ± 0.16	9.08 ± 0.16	8.95 ± 0.14	8.63 ± 0.11	8.34 ± 0.18	8.12 ± 0.06	7.71 ± 0.11	7.52 ± 0.21	7.23 ± 0.12
Brain	1	8.25 ± 0.09	8.04 ± 0.07	7.83 ± 0.18	7.77 ± 0.16	7.71 ± 0.12	7.67 ± 0.04	7.64 ± 0.09	7.48 ± 0.13	7.39 ± 0.15	7.24 ± 0.07
	2	9.05 ± 0.11	9.18 ± 0.13	9.02 ± 0.05	8.93 ± 0.08	8.64 ± 0.02	8.36 ± 0.05	7.84 ± 0.10	7.57 ± 0.16	7.33 ± 0.19	7.14 ± 0.18
Duodenum	1	8.77 ± 0.02	8.59 ± 0.07	8.29 ± 0.23	8.05 ± 0.11	7.94 ± 0.13	7.85 ± 0.07	7.81 ± 0.21	7.78 ± 0.27	7.63 ± 0.20	7.61 ± 0.19
	2	8.25 ± 0.16	8.33 ± 0.03	8.23 ± 0.07	8.16 ± 0.09	8.04 ± 0.02	7.69 ± 0.15	7.41 ± 0.19	7.26 ± 0.16	6.81 ± 0.09	6.64 ± 0.11
Rectum	1	8.68 ± 0.07	8.47 ± 0.16	8.26 ± 0.10	7.98 ± 0.23	7.94 ± 0.23	7.88 ± 0.17	7.91 ± 0.27	7.83 ± 0.14	7.74 ± 0.03	7.70 ± 0.18
	2	8.23 ± 0.05	8.27 ± 0.08	8.18 ± 0.11	8.12 ± 0.05	8.02 ± 0.10	7.78 ± 0.17	7.52 ± 0.15	7.21 ± 0.06	6.95 ± 0.09	6.78 ± 0.05
Harderian gland	1	8.32 ± 0.08	8.11 ± 0.04	7.94 ± 0.21	7.72 ± 0.14	7.62 ± 0.09	7.54 ± 0.24	7.5 ± 0.08	7.38 ± 0.22	7.41 ± 0.29	7.33 ± 0.19
	2	8.23 ± 0.15	8.35 ± 0.07	8.21 ± 0.11	8.13 ± 0.14	8.05 ± 0.19	7.77 ± 0.14	7.52 ± 0.07	7.18 ± 0.20	6.55 ± 0.01	6.39 ± 0.04
Bursa of Fabricius	1	8.86 ± 0.07	8.80 ± 0.05	8.50 ± 0.10	8.17 ± 0.09	8.02 ± 0.17	7.85 ± 0.19	7.78 ± 0.11	7.72 ± 0.09	7.6 ± 0.22	7.63 ± 0.25
	2	8.83 ± 0.11	8.92 ± 0.13	8.88 ± 0.07	8.79 ± 0.17	8.44 ± 0.19	8.25 ± 0.15	7.88 ± 0.19	7.47 ± 0.10	6.68 ± 0.08	6.53 ± 0.13

Group 1: pcDNA3.1-gC

Group 2: AHV-1 Cha strain vaccine

## Discussion

The accurate and prompt diagnosis of AHV-1 infection in waterfowls is a vital part of surveillance and disease control strategy. Currently, the diagnosis of AHV-1 usually depends on epidemiological information, clinical symptoms, pathological changes and serological methods [45-47]. However, these methods are time-consuming, inconvenient, and requiring special collection and transport conditions to maintain the viability of the virus, and the whole process may take 1 to 2 weeks. Virus can not be promptly detected from infected waterfowls with these methods. The conventional qualitative PCR method is also developed for the diagnosis of AHV-1 infection, which may not provide the sensitivity that is needed to detect low-level of viral loads. FQ-PCR is based on the conventional principles of PCR and has being become an increasingly popular way for the diagnosis of bacteria and viruses infection. The diagnostic process requires only 4 hours for detection and quantitation of bacteria and viruses from nucleic acid extraction to FQ-PCR.

The FQ-PCR assay has more advantages than conventional qualitative PCR assays, including rapidity, higher sensitivity, higher specificity, quantitive measurement, decreased risk of cross-contamination through absence of post-PCR handling and automated product detection[48]. An oligonucleotide probe of the TaqMan™ FQ-PCR assay is not included in conventional qualitative PCR, and is labelled at 5' with FAM dye as reporter and labelled at 3' with TAMRA as quencher. It facilitates highly specific binding to the targeted sequence, and results in greater accuracy in the measurement.

Previous studies have detected AHV-1 by FQ-PCR in infected ducks [35,36]. However, Yang et al. developed a relatively narrowed dynamic range for FQ-PCR, it may not be beneficial to large-scale detection in various infected cases. Guo et al. established a similar dynamic range (from 1.0 × 10^9 ^to 1.0 × 10^2 ^copies), but the end-point sensitivity (1.0 × 10^1 ^copies) was not included in the standard curve, the method may not be reliable to quantitate a low viral load (<1.0 × 10^2 ^copies). In this study, the comparisons were carried out between the established FQ-PCR method and conventional PCR method for AHV-1 detection from infected allantoic fluid, the results indicated that the established FQ-PCR method is approximately 10^3 ^times more sensitive and reliable than the conventional PCR method for clinical cases. A FQ-PCR assay was established to be highly specific for AHV-1, and had a sensitive detection limit of 1.0 × 10^1 ^DNA copies per reaction in this study, which produced excellent linear with the DNA concentration from 1.0 × 10^8 ^to 1.0 × 10^1 ^copies, with correlation coefficient of 1.000 and a reaction efficiency of 100%. The linear amplification of this assay covered a wide dynamic range suitable for quantitative applications.

The potential contamination of AHV-1 DNA that could lead to false-positive results and it was a major concern in this study. This problem was successfully avoided through the findings of high Ct values (low copies) in this assay. Furthermore, no template controls (NTCs) always be included on every plate in every experiment, which can identify the extent of pollution during the test [49]. NTCs and template controls from healthy ducks had no amplification signal in this assay, it is reasonable to think that the sample amplification is real.

The distribution and concentration of AHV-1 has been investigated in AHV-1 Cha strain-infected ducks by Qi [50]. This assay was similar with Qi's report about the distribution of the different kinds of tissues. AHV-1 attenuated vaccine can be distributed in various tissues and organs of ducks within 1 hour by subcutaneous route in this study, furthermore, the concentration of nucleic acid maintained at least 10^6 ^copies/g level at 4 weeks postinoculation. They revealed that AHV-1 attenuated vaccine can play an important role against the virulent AHV-1 in the immune ducks, but the copies of the duodenum and rectum were relatively low in infected ducks, it implyed that the various inoculate routes have large impact on the replication of vaccine virus in digestive tracts, and consistents with the gradual circulation of lymphocytes [6].

Plasmid DNA has been confirmed to widely distributed in the thymus, heart, lung, kidney, liver, mesenteric lymph nodes and other organs in a short time by intramuscular injection of DNA vaccine [51,52]. In this study, AHV-1 gC can be detected in all analyzed tissues at 1 hour postinoculation, and the concentration of gC maintained 10^7 ^copies/g level at 4 weeks postinoculation. The copies of gC in the liver, spleen and thymus were more than other tissues, and it may be due to plasmid was widely distributed in all tissues through the lymphatic flow and blood circulation in a short time [53]. These basic data can set the stage for further research about gC DNA vaccine.

Currently, the surveillance of AHV-1 becomes difficult because of the inability to differentiate the infected from vaccinated animals (DIVA). The DIVA strategy has only been recently put into practice for avian influenza virus (AIV) [54,55]. In this study, the virus loads and gC gene copy number can be accurately detected by the established FQ-PCR from inoculated ducks, because the animals were certificated as AHV-1-free by qualitative PCR assay before being infected with AHV-1 Cha and pcDNA3.1-gC. Among the different DIVA strategies, one approach is to use a DNA vaccine based on an incomplete gC gene against AHV-1. If this vaccine will be successfully constructed in the future, the developed TaqMan™ FQ-PCR assay will become perfect for the surveillance of AHV-1.

## Conclusions

In summary, the established TaqMan™ FQ-PCR was a rapid, highly specific, sensitive, repeatable and reproducible assay than conventional PCR method, and it was extremely valuable for AHV-1 detection and quantitation on the purpose of the disease transmission studies, diagnostic assays and efficacy evaluation of drugs. Also it provided some significant basic data that may be beneficial to further investigate pcDNA3.1-gC DNA vaccine. We are currently studying the dynamic distribution of gC in AHV-1-infected and DNA vaccine-inoculated ducks by using this method. We believe that this approach could expedite related AHV-1 and gC DNA vaccine research.

## Methods

### Viruses and bacteria

AHV-1 Cha strain and *Escherichia coli *JM109 were obtained from Key Laboratory of Animal Diseases and Human Health of Sichuan Province. According to the gene libraries of AHV-1 constructed by the Avian Disease Research Center of Sichuan Agricultural University[56], the pMD18-gC plasmid was obtained through a 1296 bp fragment (*gC *gene) of PCR amplification was cloned into the pMD18-T vector (Takara, Japan), and then the result of sequencing compared with the sequences of AHV-1 in GenBank. Sequence was submitted to GenBank [GenBank: EU076811] by the Avian Disease Research Center of Sichuan Agricultural University [57].

Gosling new type viral enteritis virus, duck hepatitis virus type1, duck adenovirus, goose parvovirus, Marek's disease virus, AHV-1 Chv strain virus, *Pasteurella multocida *(5: A), *Escherichia coli *(O78) and *Salmonella enteritidis *(No. 50338) were provided by Key Laboratory of Animal Diseases and Human Health of Sichuan Province. They were propagated and the nucleic acid was extracted [58-60].

### Standard templates preparation

The purified gC gene was obtained from pMD18-gC by using restriction enzymes (*Eco*R I and *Xho *I) (Takara, Japan), and was inserted into the eukaryotic expression vector pcDNA3.1(+) (Invitrogen, USA) according to the manufacturer's protocol. The constructed pcDNA3.1-gC plasmid was transformed into *Escherichia coli *JM109 cells. pcDNA3.1-gC plasmid was extracted by TIANprep plasmid extraction kit (Tiangen, China) according to manufacturer's protocol. The presence of target DNA was confirmed by PCR amplification with primers P1 and P2 (generated by Takara, Japan) targeting the *gC *gene on a Mycycler™ thermo cycler system (Bio-Rad, USA), their sequences were listed in Table 3. The product size was 1296 bp. DNA sequencing showed that pcDNA3.1-gC is real.

**Table 3:** Oligonucleotide sequences of primers and probe used in AHV-1 FQ-PCR detection

Name	Type	Sequences (5' to 3')		Length (nt)	Amplicon size (bp)
P1	Forward	CGGAATTCCAAAACGCCGCACAGATGAC		28	1296
P2	Reverse	CCCTCGAGGTATTCAAATAATATTGTCTGC		30	
P3	Forward	GAAGGACGGAATGGTGGAAG		20	78
P4	Reverse	AGCGGGTAACGAGATCTAATATTGA		25	
P	Probe	FAM-CCAATGCATCGATCATCCCGGAA-TAMRA		23	

The underlined sequences of P1 and P2 are *Eco*R I and *Xho *I restriction sites respectively

### PCR primers and probe design

The FQ-PCR assay primers and TaqMan™ probe (named P3, P4 and P respectively, generated by Genecore Corporation, China) design was carried out by using the Primer Express™ software supplied by Applied Biosystems according to the sequence of *gC *gene [GenBank: EU076811] and their sequences were listed in Table 3. The forward and reverse primers amplified a 78 bp fragment of AHV-1 *gC *gene. The fluorogenic probe was labelled at 5' with FAM (6-carboxyfluorescein) dye as reporter and labelled at 3' with TAMRA (tetra-methylcarboxyrhodamine) as quencher.

### Protocol optimization

FQ-PCR was performed in an iCycler iQ Multicolor Real-Time PCR Detection System (Bio-Rad, USA) with a reaction mixture (20 μL) containing 10 μL 2 × Premix Ex Taq™ (Takara, Japan) and 2 μL standard template according to the manufacturer's protocol. Autoclaved double-filtered nanopure water was added to get the final volume to 20 μL. The reactions were optimized in triplicate based on primers (P3 and P4) and TaqMan™ probe (P) concentration selection criteria, which was performed according to 5 × 5 matrix of primers concentrations (0.2, 0.3, 0.4, 0.5 and 0.6 μmol/L) and probe concentrations (0.1, 0.2, 0.25, 0.3 and 0.35 μmol/L). The two-step PCR cycling condition as follows: initial denaturation and hot-start Taq DNA polymerase activation at 95°C for 5 min, 45 cycles of denaturation at 94°C for 5 s, primer annealing and extension at 53°C for 30 s with fluorescence acquisition during each annealing and extension stage. The tests were carried out by using the 0.2 mL PCR tubes (Axygen, USA).

### standard curve establishment

The recombinant plasmid pcDNA3.1-gC was used to establish standard curve as standard DNA of FQ-PCR. pcDNA3.1-gC concentration was determined by taking the absorbance at 260 nm by using a Smartspec 3000 spectrophotometer (Bio-Rad, USA) and purity was confirmed by using the 260/280 nm ratio. The pcDNA3.1-gC copies/μL was calculated and the purified plasmid DNA was serially diluted 10-fold in TE buffer, pH 8.0, from 5.0 × 10^7 ^to 5.0 × 10^0 ^plasmid copies/μL. The Primers (P3 and P4) were used for this amplification, These dilutions were used as amplification standards to construct the standard curve by plotting the plasmid copy number logarithm against the Ct values under optimum conditions. The standard curve and its correlation coefficient were generated through the software of iCycler IQ Detection System (Bio-Rad, USA) according to the manufacturer's protocol.

### Amplification sensitivity, specificity, repeatability and reproducibility

The sensitivity of the assay was used as the limit extent of detection when testing 10-fold diluted DNA standards in triplicate. The dilution of plasmid pcDNA3.1-gC was ranging from 1.0 × 10^5 ^to 1.0 × 10^0 ^copies/reaction. This test was performed under optimum conditions.

The different 40 liver samples had been confirmed positive for AHV-1 by using the conventional PCR from infected ducks, these samples were retested with the established FQ-PCR method to evaluate the sensitivity of this method for clinical cases.

AHV-1 Cha strains was propagated in the allantoic cavity of 10-day-old SPF duck embryo. The allantoic fluid was harvested from dead embryo. Viral DNA from allantoic fluid was extracted by using TIANamp viral Genomic (DNA/RNA) extracting kit (Tiangen, China) according to the manufacture's instructions, then examined by the established FQ-PCR method and conventional PCR under same circumstance in triplicate after it was 10-fold diluted with sterile ultrapure water. The detection limit of the FQ-PCR was determined based on the highest dilution that resulted in the presence of Ct value in real-time PCR detection. The detection limit of the conventional PCR was determined through the highest dilution that resulted in the presence of clear amplified fragments (78 bp) on the agarose gel. The end-point sensitivity of both assays were calculated.

The specificity of the assay was evaluated by testing the different kinds of templates including pcDNA3.1-gC, AHV-1 Cha, AHV-1 Chv, gosling new type viral enteritis virus, duck hepatitis virus type1, duck adenovirus, goose parvovirus, Marek's disease virus, *Pasteurella multocida *(5: A), *Escherichia coli *(O78) and *Salmonella enteritidis *(No. 50338), then the liver DNA of the healthy duck should be added in this experiment as a negative control.

In order to assess intra-assay variability, eight dilutions of pcDNA3.1-gC (1.0 × 10^8^-1.0 × 10^1 ^copies/reaction) were prepared separately. These samples were assayed simultaneously in triplicate in a same experiment. Five experiments were performed on different days in order to assess inter-assay variability, using eight pcDNA3.1-gC dilutions (1.0 × 10^8^-1.0 × 10^1 ^copies/reaction). All tests were performed under optimum conditions. The mean Ct values, SD values and CV values were calculated independently for each DNA dilution.

### Detection of AHV-1 *gC *gene in samples for practical applications

This study was conducted with 90 AHV-1-free Peking ducks (28 days old) from a AHV-1-free farm which were certificated with qualitative PCR as described by Song[61]. 60 ducks were randomly divided into two equal groups in this study (30 in each group). Thirty non-immunized ducks in Groups 3 used as controls. Ducks in Groups 1-2 were inoculated with 200 μg pcDNA3.1-gC (2.714 × 10^13 ^copies) as DNA vaccine by intramuscular route and with 0.2 mL AHV-1 Cha strain vaccine (6.692 × 10^11 ^copies) by subcutaneous route respectively. At each of ten sampling times, three vaccinated ducks of each immune group were chosen randomly for sampling. The liver, pancreas, spleen, kidney, lung, thymus, heart, brain, duodenum, rectum, Harderian gland and bursa of Fabricius were collected at 1 h, 4 h, 8 h, 12 h, 1 d, 3 d, 5 d, 7 d, 2 wk and 4 wk postinoculation respectively. DNA of all these samples were extracted by Animal cell/tissue DNA magnetic bead extraction kit (Bioeasy Technology, China) in Thermo Scientific KingFisher (mL) (Thermo, USA) from 15 mg tissues according to the manufacturer's protocol, followed by being dissolved in 50 μL sterile ultrapure water. Then, 2 μL DNA of each sample was prepared to detect AHV-1 gC accumulation in triplicate by TaqMan™ FQ-PCR.

## Competing interests

The authors declare that they have no competing interests.

## Authors' contributions

QZ and KS carried out most of the experiments. QZ drafted the manuscript. AC and MW strictly revised the manuscript and the experiment design. CX, DZ, RJ, QL, YZ, ZC and XC assisted with the experiments. All of the authors read and approved the final manuscript.
